# *Yuebeipotamon calciatile*, a new genus and new species of freshwater crab from southern China (Crustacea, Decapoda, Brachyura, Potamidae)

**DOI:** 10.3897/zookeys.615.9964

**Published:** 2016-09-07

**Authors:** Chao Huang, Hsi-Te Shih, Si Ying Mao

**Affiliations:** 1 Palaeontology, Geobiology and Earth Archives Research Centre, School of Biological, Earth and Environmental Sciences, UNSW, Kensington, NSW 2052, Australia; 2 Australian Museum, 6 College Street, Sydney, NSW 2010, Australia; 3 School of Life Sciences, Sun Yat-sen University, Guangzhou 510275, China; 4 Department of Life Science, National Chung Hsing University, 250, Kuo Kuang Road, Taichung 402, Taiwan

**Keywords:** China, freshwater crabs, new genus, new species, Potamidae, systematics, Yuebeipotamon calciatile, 16S rDNA

## Abstract

A new genus and species of freshwater crab, *Yuebeipotamon calciatile* **gen. n., sp. n.**, is described from southern China. While the carapace features are superficially similar to species of *Sinopotamon* Bott, 1967, *Longpotamon* Shih, Huang & Ng, 2016, and *Tenuilapotamon* Dai, Song, Li, Chen, Wang & Hu, 1984, the new genus possesses a distinctive combination of carapace, ambulatory leg, male thoracic sternal, male abdominal, and gonopodal characters that distinguish it from these and other genera. Molecular evidence derived from the mitochondrial 16S rDNA supports the establishment of a new genus.

## Introduction

The South China region is diverse in freshwater crabs from the family Potamidae Ortmann, 1893. Despite its large land mass, Guangdong has a relatively low diversity when compared to other South Chinese provinces (Dai 1999, Shih and Ng 2011), which is probably the result of insufficient surveys conducted in this region.

In the past few years, there has been a growing trend in the aquarium trade for colorful freshwater crabs from South China, with species from the genera *Nanhaipotamon* Bott, 1968, *Hainanpotamon* Dai, 1995, *Neilupotamon* Dai & Türkay, 1997, and *Heterochelamon* Dai & Türkay, 1997, sometimes showing up in pet shops and even exported to other countries. We initially obtained one such species from the trade that has relatively long ambulatory legs with reddish to purplish coloration, which was interesting as it possessed a unique male first gonopod structure. The native ornamental fish dealer who sold these crabs to us eventually agreed with the first author’s request to conduct a survey at his collection site, which was in northern Guangdong. This new species was compared to all known genera from around the region and while superficially similar to *Sinopotamon* Bott, 1967, *Longpotamon* Shih, Huang & Ng, 2016, and *Tenuilapotamon* Dai, Song, Li, Chen, Wang & Hu, 1984, in general carapace morphology (Dai 1999, Shih et al. 2016), it can immediately be differentiated by distinctive combinations of carapace, ambulatory leg, male thoracic sternal, male abdominal and gonopod characters. A molecular analysis conducted using the mitochondrial 16S rRNA marker also suggests that it does not belong to any known genera. Therefore, a new genus is established in this paper for this new species.

## Material and methods

Specimens were collected from Yingde City of northern Guangdong, preserved in 75% ethanol and have been deposited in the Sun Yat-sen Museum of Biology, Sun Yat-sen University (SYSBM), Guangzhou, China, and the National Zoological Museum of China, Institute of Zoology, Chinese Academy of Sciences
(IZCAS), Beijing, China. Measurements, in millimeters, are for the carapace width and carapace length. The following abbreviations are used: G1 – male first gonopod; G2 – male second gonopod.

Genomic DNA was isolated from the muscle tissue of ambulatory legs by using the Tiangen universal DNA purification kit (Beijing, China) and GeneMark tissue and cell genomic DNA purification kit (Taichung, Taiwan). A region of ~550 basepairs (= bp) of the 5’-end of the 16S gene was selected for amplification with polymerase chain reaction (PCR) using the primers 1471 and 1472 (Crandall and Fitzpatrick 1996). The PCR conditions were denaturation for 45 s at 94 °C, annealing for 40 s at 45 °C, and extension for 120 s at 72 °C (35 cycles), followed by extension for 10 min at 72 °C. Sequences were obtained by automated sequencing (Applied Biosystems 3730) and were aligned with the aid of ClustalW (vers. 1.4, Thompson et al. 1994), after verification with the complementary strand. To confirm the systematic position of this species, the 16S sequences of genera from the eastern Asian continent in Shih et al. (2009), as well as the more recently described genus *Minutomon* Huang, Mao & Huang, 2014, were included for comparison. Sequences of the haplotypes have been deposited in a DNA Data Bank of Japan (DDBJ). We followed Shih et al. (2009) to exclude the variable regions in loop regions of the 16S which could not be aligned adequately for phylogenetic analyses.

The best-fitting model for sequence evolution of the 16S dataset was determined by MrModeltest (vers. 2.2, Nylander 2005), selected by the Akaike information criterion (AIC). The best model obtained was HKY+I+G, and was subsequently applied for Bayesian inference (BI) and maximum likelihood (ML) analyses. The BI analysis was performed with MrBayes (vers. 3.2.2, Ronquist et al. 2012) and the search was run with four chains for 10 million generations, with trees sampled every 1000 generations. The convergence of chains was determined by the effective sample size (ESS) (>200 as recommended) in Tracer (vers. 1.5, Rambaut and Drummond 2009) and the first 1000 trees were discarded as the burnin (determined by the average standard deviation of split frequency values below the recommended 0.01; Ronquist et al. 2005). ML analysis was conducted in GARLI (vers. 2.0, Zwickl 2006), with 10 replicate searches (searchreps = 10) and 100 bootstraps (bootstrapreps = 100) and the consensus tree from the GARLI output was computed using the program PAUP* (vers. 4.0b10, Swofford 2003) to assess node supports.

## Systematic account

### Family Potamidae Ortmann, 1896

#### Yuebeipotamon gen. n.

Taxon classificationPlantaeDecapodaPotamidae

http://zoobank.org/BF003144-6BF4-43AD-8772-DDF577DD6F22

##### Diagnosis.

Carapace subquadrate, with dorsal surface slightly convex, surface generally smooth, rugose on anterolateral regions (Fig. 2A); postorbital and epigastric cristae distinct, not confluent (Fig. 2A); external orbital angle sharply triangular, separated from anterolateral margin by a narrow gap (Fig. 2A, B); median lobe of posterior margin of epistome sharply triangular (Fig. 2B); third maxilliped with relatively broad ischium, exopod of third maxilliped reaches beyond anterior edge of ischium, with short flagellum (Fig. 3D); male abdomen triangular, with short triangular telson (Fig. 2C); G1 generally slender, terminal segment large, elongated, with subbasal flap (Figs 2D, 3B, C); basal segment of G2 subquadrate (Fig. 3A).

##### Type species.

*Yuebeipotamon calciatile* sp. n., by monotypy.

##### Etymology.

The genus name is derived from the Chinese spelling system “Yue Bei”, which means northern Guangdong, for the locality of this genus. The suffix “Potamon” refers to the type genus of the family Potamidae, *Potamon*. Gender of genus neuter.

##### Remarks.

Although *Sinopotamon*, *Longpotamon*, *Tenuilapotamon*, and *Yuebeipotamon* are superficially similar in carapace features, *Yuebeipotamon* can easily be distinguished from by a number of characters (Table 1).

**Table 1.** Morphological differences among *Yuebeipotamon* gen. n., *Tenuilapotamon* Dai, Song, Li, Chen, Wang & Hu, 1984, *Sinopotamon* Bott, 1967, and *Longpotamon* Shih, Huang & Ng, 2016.

Character	*Yuebeipotamon*	*Tenuilapotamon*	*Sinopotamon*	*Longpotamon*
Epibranchial teeth	distinct, sharp (Fig. 2A)	indistinct, granular (cf. Dai 1999: pl. 29)	varied (cf. Dai 1999: pl. 17)	varied (cf. Zou et. al. 2008: fig. 1)
Ambulatory legs	slender (Fig. 2A)	slender (cf. Dai 1999: pl. 29)	stout (cf. Dai 1999: pl. 17)	stout (cf. Zou et al. 2008: fig. 1)
Size of triangular structure of male thoracic sternites 1, 2	relatively large (Fig. 4C)	relatively small (unpublished data)	relatively small (cf. Shih et al. 2016: fig. 3C)	relatively small (cf. Shih et al. 2016: fig. 6C)
Male telson	relatively short, triangular (Fig. 2C)	relatively long, subtriangular (cf. Dai 1999: fig. 225)	relatively long, nipple shaped (cf. Dai 1999: fig. 139)	relatively long, subtriangular (cf. Zou et al. 2008: fig. 2)
G1	long, reaching beyond tubercle of abdominal lock (Fig. 2D)	short, not reaching tubercle of abdominal lock (cf. Dai 1999: fig. 225)	long, reaching beyond tubercle of abdominal lock (cf. Dai 1999: fig. 139)	short, not reaching tubercle of abdominal lock (cf. Dai 1999: fig. 137)
Terminal segment of G1	lo006Ecxcg, with subbasal flap (Fig. 3C)	short, without subbasal flap (cf. Dai 1999: fig. 225)	short, without subbasal flap (cf. Dai 1999: fig. 139)	short, without subbasal flap (cf. Zou et al. 2008: fig. 2)

##### Comparative material from China.

*Sinopotamon kwanhsiense* Tai & Sung, 1975: 1 ♂ (45.3 × 36.0 mm), IZCAS CB7659, Chengdu, Sichuan, 1984. *Sinopotamon pingshanense* Dai & Liu, 1994: 1 ♂ (45.2 × 34.3 mm), IZCAS CB8278, Muchuan, Sichuan, Oct. 1986. *Longpotamon anyuanense* (Dai, Zhou & Peng, 1995): 1 ♂ (45.7 × 39.1 mm) (SYSBM 001080), Shaoguan City, Guangdong, Apr. 2013; *Longpotamon chekiangense* (Tai & Sung, 1975): 1 ♂ (32.0 × 26.1 mm) (SYSBM 001079), Lucheng District, Wenzhou City, Zhejiang, Mar. 2013; *Longpotamon fukienense* (Dai & Chen, 1979): 1 ♂ (45.7 × 39.1 mm) (SYSBM 001054), Fuqing City, Fujian, May 2013. *Tenuilapotamon joshuiense* (Dai, Song, He, Cao, Xu & Zhong, 1975): 1 ♂ (23.6 × 19.3 mm) (SYSBM 001270), Lianyuan City, Hunan, Sep. 2013; 1 ♀ (23.7 × 19.4 mm) (SYSBM 001271), same data as above.

#### Yuebeipotamon calciatile sp. n.

Taxon classificationPlantaeDecapodaPotamidae

http://zoobank.org/7DF2B1CA-5E8A-42D1-837D-B35EDA68B10C

Figs 1 , 2 , 3 , 4

##### Material examined.

Holotype: ♂ (32.4 × 27.0 mm) (SYSBM 001294), Yingde, Guangdong, China, karstic hillstream, coll. C. Huang, Jun. 2014. Paratypes: 1 ♀ (allotype) (33.0 × 27.9 mm) (SYSBM 001295), same data as holotype; 2 ♂♂ (40.6 × 32.5 mm, 41.0 × 32.9 mm) (SYSBM 001296, 001297), same data as holotype; 1 ♂ (37.9 × 31.8 mm) (IZCAS), same data as holotype. Others: 3 ♂♂ (38.3 × 31.7 mm, 36.5 × 29.9 mm, 20.7 × 17.4 mm) (SYSBM 001298, 001299, 001300), Yingde, Guangdong, China, karstic hill stream, coll. G.-H. Yuan, May 2014; 2 ♀♀ (17.1 × 14.2 mm, 18.2 × 14.8 mm) (SYSBM 001301, 001302), same data as above.

##### Diagnosis.

As for genus.

##### Description.

Carapace subquadrate; dorsal surface slightly convex transversely, longitudinally; surface with rugose on anterolateral region (Fig. 2A). Front slightly deflexed, margin almost straight on dorsal view (Fig. 2A). Epigastric cristae low, separated by narrow gap (Fig. 2A, B). Postorbital cristae blunt, laterally expanded, not fused with epigastric cristae or reach the anterolateral margin (Fig. 2A, B). Branchial regions slightly convex (Fig. 2A). Cervical groove shallow, inconspicuous (Fig. 2A). Mesogastric region slightly convex (Fig. 2A). External orbital angle sharply triangular (Fig. 2A). Epibranchial tooth pointed, distinct (Fig. 2A, B). Anterolateral margin distinctly cristate, lined with approximately 17–19 granules; lateral part bent inwards (Fig. 2A). Posterolateral margin comparatively smooth, lined with multiple oblique striae, converging towards posterior carapace margin (Fig. 2A). Orbits large; supraorbital and infraorbital margins cristate, lined with numerous inconspicuous granules (Fig. 2B). Suborbital, subhepatic and upper parts of pterygostomial regions covered with rounded granules (Fig. 2B). Third maxilliped with merus about 1.1 times as broad as long; ischium about 1.5 times as long as broad; merus trapezoidal, with median depression; ischium trapezoidal, with distinct median sulcus; exopod reaching to proximal third of merus, with short flagellum reaching proximal three-fifths width of merus; upper-inner margin of ischium forming subauriculiform structure (Figs 2B, 3D). Posterior margin of epistome narrow; median lobe sharply triangular, lateral margins almost straight (Fig. 2B).

**Figure 1.**
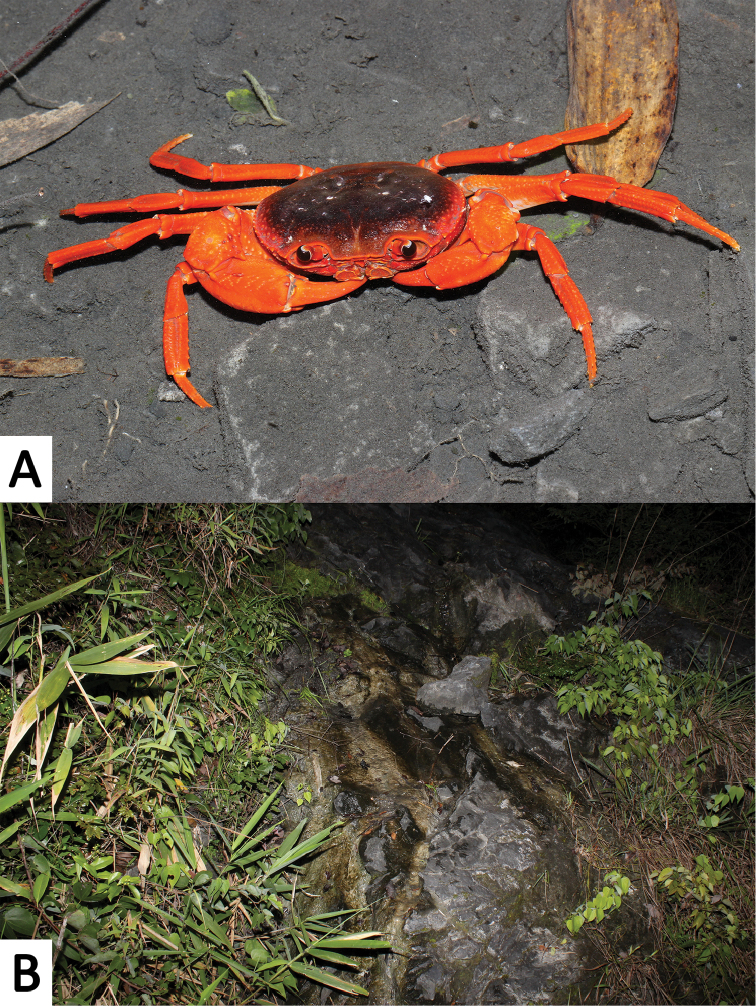
*Yuebeipotamon calciatile* gen. n., sp. n., color in life. **A** male, specimen not collected **B** a limestone hill stream at the type locality.

**Figure 2.**
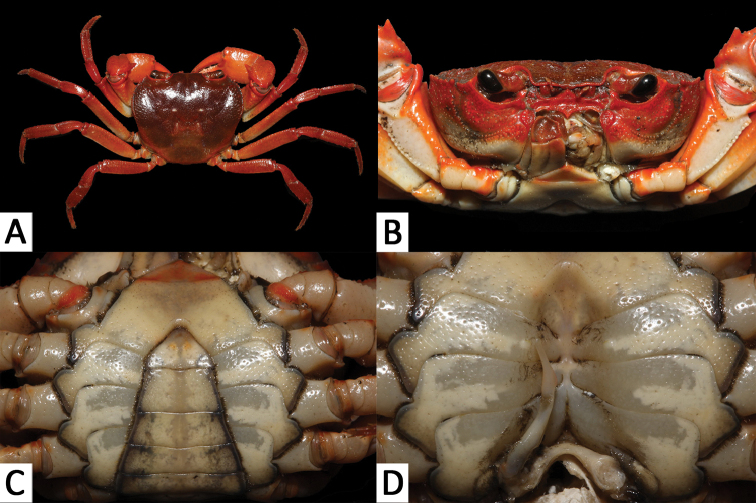
*Yuebeipotamon calciatile* gen. n., sp. n., male holotype (32.4 × 27.0 mm) (SYSBM 001294). **A** dorsal view **B** frontal view of carapace **C** ventral view showing anterior thoracic sternum and abdomen **D** ventral view showing sterno-abdominal cavity with right G1 in situ (left G1 removed).

**Figure 3.**
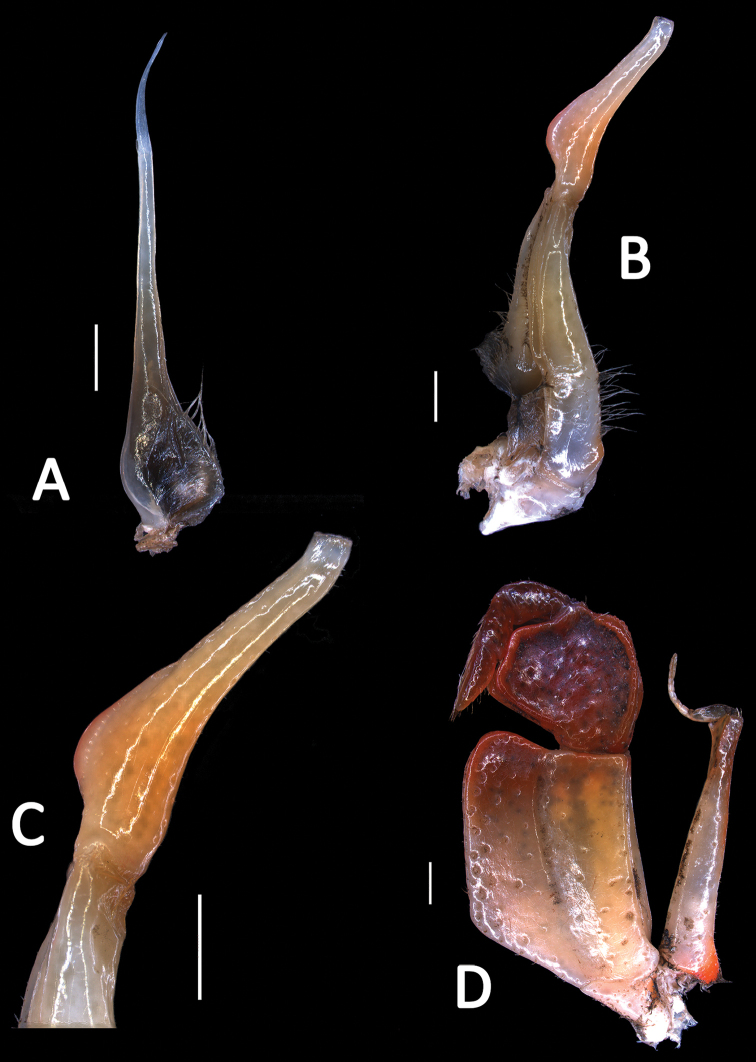
*Yuebeipotamon calciatile* gen. n., sp. n., male holotype (32.4 × 27.0 mm) (SYSBM 001294). **A** left G2 **B** left G1 (ventral view) **C** terminal segment of G1 (ventral view) **D** left third maxilliped. Scale bar 1.0 mm.

Chelipeds unequal (Fig. 2A). Merus cross-section trigonal; margins crenulated, dorsal-outer surface granulated (Fig. 2B). Carpus with sharp spine at inner-distal angle, spinule at base, dorsal surface with curved striae (Fig. 2A). Palm of larger chela about 1.6 times as long as high. Movable finger equal to fixed finger (Fig. 2A). Inner margin of fingers with rounded, blunt teeth; with small gap when fingers closed.

Ambulatory legs relatively slender, surfaces generally smooth (Fig. 2A). Last leg with propodus about 2.5 times as long as board, approximately same length as dactylus (Fig. 2A).

**Figure 4.**
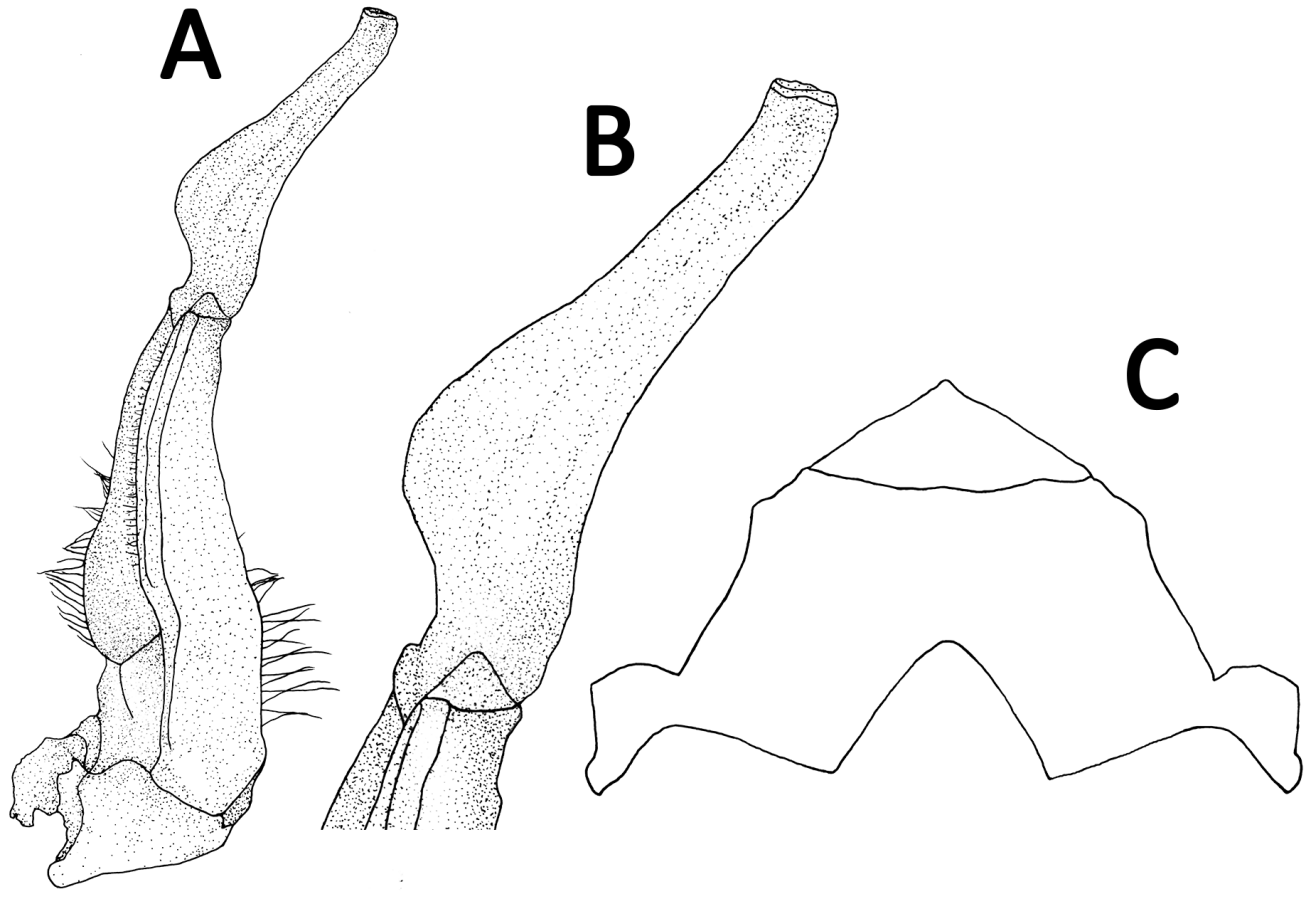
*Yuebeipotamon calciatile* gen. n., sp. n., male holotype (32.4 × 27.0 mm) (SYSBM 001294). **A** left G1 (ventral view) **B** terminal segment of G1 (ventral view) **C** anterior thoracic sternum.

Male thoracic sternum generally smooth, weakly pitted; sternites 1, 2 completely fused to form triangular structure; sternites 2, 3 separated by continuous suture; sternites 3, 4 fused without obvious median suture; male sterno-abdominal cavity reaching to imaginary line joining median part of coxae of cheliped; median longitudinal groove between sternites 7, 8 deep (Figs 2C, 4C).

Male abdomen narrowly triangular; somites 3–6 progressively broader longitudinally; somite 6 about 1.9 times as board as long; telson about 1.5 times as board as long with a rounded tip, lateral margins of telson slightly concave (Fig. 2C).

G1 generally slender; terminal segment large, elongated, inner margin with subbasal flap; tip of terminal segment reaches beyond tubercle of abdominal lock in situ; distal part of subterminal segment relatively narrow; subterminal segment about 1.3 times as long as terminal segment (Fig. 2D, 3B, C). G2 basal segment about 2.8 times length of flagelliform distal segment (Fig. 3A).

##### Variation.

Adult specimens are usually much more brightly colored than juveniles. The terminal segment of the G1 may vary in proportionate length, while the angle at which it points varies from around 45–60 degrees.

##### Etymology.

The species name, “*calciatile*”, means living on limestone, relating to its natural habitat.

##### Color.

Carapace is usually maroon to dark brown, while chelipeds and ambulatory legs are reddish to purplish in life (Fig. 1A).

##### Ecology.

This primarily aquatic species is found in the pools of limestone hill streams where they hide in crevices. Almost each pool was occupied by at least one crab at the type locality, which is a relatively high density of distribution. Its slender legs indicate that this species has good climbing abilities and mobility on land. These abilities are assumed to be advantageous in the volatile and short-lived nature of limestone hill streams, which may force them to intermittently find new water sources. No other potamids were observed at the type locality.

### DNA analyses and discussion

**Figure 5.**
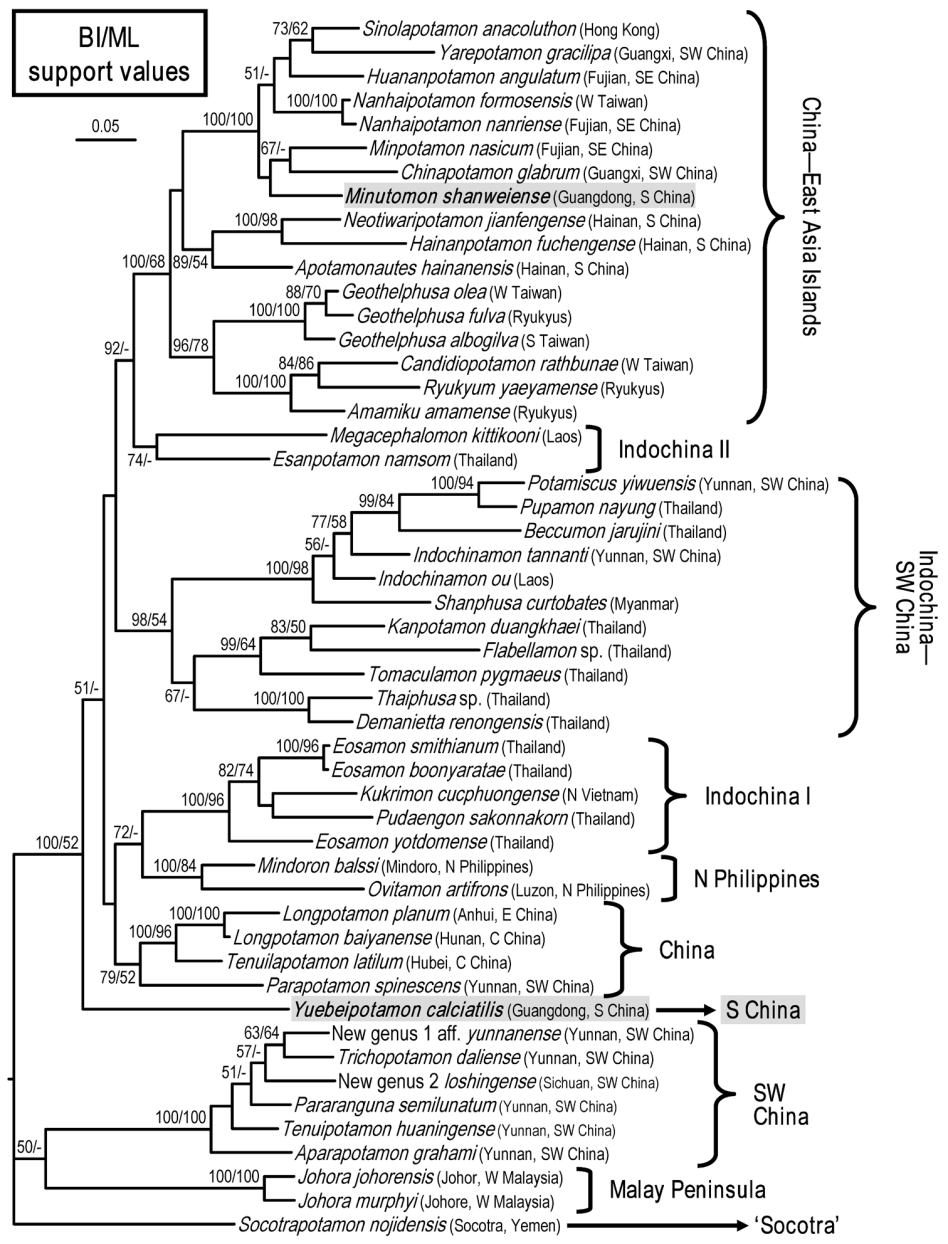
A Bayesian inference (BI) tree of 16S rDNA for the subfamily Potamiscinae, with the sequences in Shih and Ng (2009), as well as *Yuebeipotamon* gen. n. and *Minutomon* Huang, Mao & Huang, 2014 (gray highlighted). Probability values at the nodes represent support values for BI and maximum likelihood (ML). Only values > 50% are shown.

In total, 51 species from 44 genera of potamids were included in the phylogenetic analyses. A 503 bp segment, excluding the variable regions, of the 16S rDNA was amplified and aligned. The accession numbers of the 16S sequences of *Yuebeipotamon calciatile* sp. n. and *Minutomon shanweiense* Huang, Mao & Huang, 2014 are LC176064 and LC176065, respectively. The phylogenetic tree of the 16S was reconstructed using BI analysis, with support values from ML analysis (Fig. 5). The tree strongly indicates that *Yuebeipotamon* does not belong to any one of the genera included in this study, giving support to the current taxonomic treatment, i.e. it is a new genus. From its basal position to most known genera from East Asia and Southeast Asia, it suggests that this genus might be from an ancient lineage. However, *Yuebeipotamon* is distributed in Guangdong Province, part of the Pearl River Basin, which is thought to have younger lineages due to its distance from the proposed center of origin for the Potamidae, Yunnan Province (Shih and Ng 2011). This indicates that the ancestor for the genus may have dispersed to the eastern regions of China earlier than previously thought. More genetic markers are necessary to reveal the exact relationship of this genus. In addition, the recently established *Minutomon* (see Huang et al. 2014) is also supported genetically and belongs to the “China-East Asia Islands” clade which is closely related to genera from continental China (Shih et al. 2009).

## Supplementary Material

XML Treatment for Yuebeipotamon

XML Treatment for Yuebeipotamon calciatile

## References

[B1] Crandall KA Fitzpatrick JFJ (1996) Crayfish molecular systematics: using a combination of procedures to estimate phylogeny. Systematic Biology 45: 1–26. doi: 10.1093/sysbio/45.1.1

[B2] Dai AY (1999) Fauna Sinica: ArthropodaCrustaceaMalacostracaDecapodaParathelphusidaePotamidae. Science Press, Beijing, China, 501 pp [In Chinese with English summary]

[B3] Huang C Mao SY Huang JR (2014) Two new potamid crabs, *Yuexipotamon arcophallus* new genus, new species and *Minutomon shanweiense* new genus, new species, (Crustacea: Decapoda: Brachyura: Potamidae) from southern China. Zootaxa 3764: 455–466. doi: 10.11646/zootaxa.3764.4.5 24870647 10.11646/zootaxa.3764.4.5

[B4] Nylander JAA (2005) MrModeltest version 2.2. Program distributed by the author Evolutionary Biology Centre, Uppsala University, Uppsala, Sweden.

[B5] Rambaut A Drummond AJ (2009) Tracer, Version 1.5. http://beast.bio.ed.ac.uk/Tracer

[B6] Ronquist F Huelsenbeck JP van der Mark P (2005) MrBayes, ver. 3.1. http://mrbayes.csit.fsu.edu/manual.php

[B7] Ronquist F Teslenko M van der Mark P Ayres DL Darling A Höhna S Larget B Liu L Suchard MA Huelsenbeck JP (2012) MRBAYES 3.2: efficient Bayesian phylogenetic inference and model choice across a large model space. Systematic Biology 61: 539–542. doi: 10.1093/sysbio/sys029 22357727 10.1093/sysbio/sys029 PMC3329765

[B8] Shih HT Huang C Ng PKL (2016) A re-appraisal of the widely-distributed freshwater crab genus *Sinopotamon* Bott, 1967, from China, with establishment of a new genus (Crustacea: Decapoda: Potamidae). Zootaxa 4138: 309–331. doi: 10.11646/zootaxa.4138.2.5 27470766 10.11646/zootaxa.4138.2.5

[B9] Shih HT Ng PKL (2011) Diversity and biogeography of freshwater crabs (Crustacea: Brachyura: Potamidae, Gecarcinucidae) from East Asia. Systematics and Biodiversity 9: 1–16. doi: 10.1080/14772000.2011.554457

[B10] Shih HT Yeo DCJ Ng PKL (2009) The collision of the Indian plate with Asia: molecular evidence for its impact on the phylogeny of freshwater crabs (Brachyura: Potamidae). Journal of Biogeography 36: 703–719. doi: 10.1111/j.1365-2699.2008.02024.x

[B11] Swofford DL (2003) PAUP*: Phylogenetic Analysis Using Parsimony (*and Other Methods), version 4. Sinauer Associates, Sunderland, Massachusetts.

[B12] Thompson JD Higgins DG Gibson TJ (1994) CLUSTAL W: improving the sensitivity of progressive multiple sequence alignment through sequence weighting, position-specific gap penalties and weight matrix choice. Nucleic Acids Research 22: 4673–4680. doi: 10.1093/nar/22.22.4673 7984417 10.1093/nar/22.22.4673 PMC308517

[B13] Zou JX Naruse T Zhou XM (2008) On a new species of freshwater crab of the genus *Sinopotamon* (Decapoda, Brachyura, Potamidae) from Wuyi mountain, southeastern China. Crustaceana 81: 1381–1387. doi: 10.1163/156854008X361076

[B14] Zwickl DJ (2006) Genetic Algorithm Approaches for the Phylogenetic Analysis of Large Biological Sequence Datasets under the Maximum Likelihood Criterion. Ph.D. Dissertation University of Texas at Austin, Austin, Texas.

